# Invasive Aspergillus Tracheobronchitis Presenting as Subglottic Stenosis: A Case Report

**DOI:** 10.1002/rcr2.70518

**Published:** 2026-03-01

**Authors:** Takaya Sato, Kentaro Minegishi, Masatomo Miyata, Osuga Fumie, Keigo Sudo, Masaya Sogabe, Mitsuru Maki, Katsuyuki Yoshida, Takeshi Yamashita, Takahiko Fukuchi, Shunsuke Endo, Hiroyoshi Tsubochi

**Affiliations:** ^1^ Department of General Thoracic Surgery Jichi Medical University Saitama Medical Center Saitama Japan; ^2^ General Medicine Jichi Medical University Saitama Medical Center Saitama Japan

## Abstract

Invasive *Aspergillus* tracheobronchitis (IATB) is a rare but potentially fatal manifestation of invasive aspergillosis. Because of its high mortality, prompt recognition and initiation of antifungal therapy are crucial. We report the case of a 71‐year‐old woman with a history of diffuse large B‐cell lymphoma (DLBCL) who presented with progressive dyspnea. Computed tomography revealed subglottic airway stenosis, necessitating an emergency tracheostomy to secure the airway. Histopathological and microbiological examination of bronchoscopic biopsy specimens demonstrated infiltration of *Aspergillus fumigatus*, establishing the diagnosis of IATB. Antifungal therapy with voriconazole was promptly initiated, resulting in gradual clinical and endoscopic improvement. The tracheostomy tube was successfully removed, and the airway lesion showed complete resolution. This case underscores the importance of early diagnosis and timely antifungal therapy to achieve a favourable outcome. When severe airway obstruction is present, airway interventions such as tracheostomy may be lifesaving.

## Graphical abstract

We report a case of subglottic stenosis due to invasive *Aspergillus* tracheobronchitis requiring tracheostomy, highlighting the clinical importance of early diagnosis in airway‐threatening fungal infections.

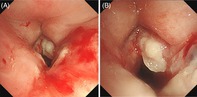

## 1 Introduction

Invasive aspergillosis most often occurs in immunocompromised patients and is associated with high morbidity and mortality [1]. The infection typically begins after inhalation of fungal spores, leading primarily to pulmonary parenchymal involvement. In contrast, IATB is a rare manifestation of invasive pulmonary aspergillosis, characterised by direct invasion of the tracheobronchial tree rather than parenchymal lung.

Importantly, IATB may present with nonspecific respiratory symptoms or upper‐airway obstruction, and radiographic findings can be absent, leading to delayed diagnosis. When the disease involves the subglottic or upper airway, progressive stenosis may rapidly result in life‐threatening airway compromise. Because such presentations can mimic other causes of subglottic stenosis, including malignancy or post‐intubation injury, IATB represents a common diagnostic pitfall in immunocompromised patients [2].

Therefore, in patients with immunosuppression who present with unexplained subglottic or upper‐airway stenosis, early bronchoscopic evaluation with tissue biopsy should be strongly considered to establish a definitive diagnosis and initiate timely antifungal therapy. We herein report a case of subglottic stenosis due to invasive *Aspergillus* tracheobronchitis requiring tracheostomy, highlighting the clinical importance of early diagnosis in airway‐threatening fungal infections.

## 2 Case Report

A 71‐year‐old woman presented to our hospital with progressive dyspnea. On examination, audible inspiratory stridor was noted. Her medical history was notable for diffuse large B‐cell lymphoma (DLBCL), which had achieved complete remission 15 years earlier following R‐CHOP (rituximab, cyclophosphamide, doxorubicin, vincristine, prednisolone) therapy. One year prior to this presentation, she experienced a recurrence involving the thoracic spine and was treated with Pola‐BR (polatuzumab, bendamustine, rituximab), again achieving remission. She had no history of smoking or alcohol use.

**FIGURE 1:**
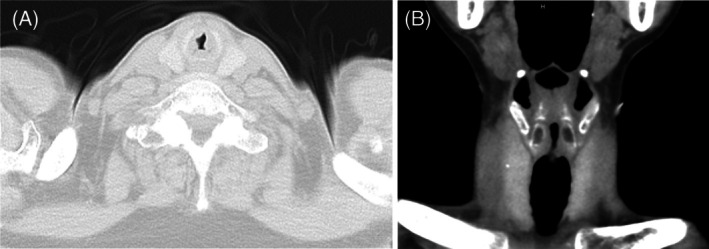
Computed tomography reveals airway stenosis due to thickening of the subglottic lesion: (A) axial view, (B) coronal view. No pulmonary parenchymal involvement is observed.

**FIGURE 2:**
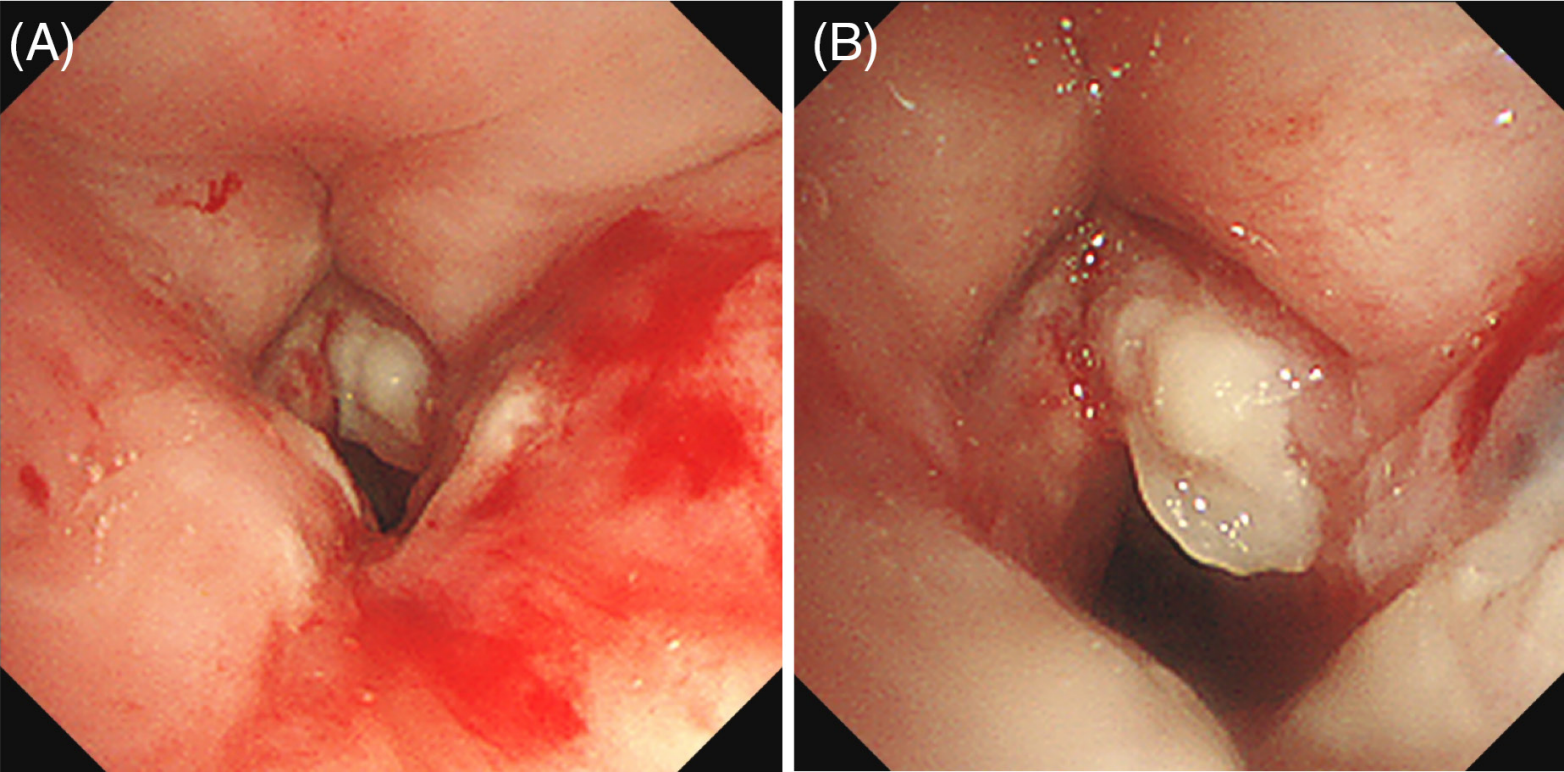
Initial bronchoscopy revealed an ulcerative lesion with white plaque and stenosis in the subglottic and vocal cords: (A) vocal cord level, (B) subglottic level.

Computed tomography (CT) of the neck and chest demonstrated subglottic stenosis without evidence of pulmonary parenchymal involvement (Figure 1). To prevent impending airway obstruction, an emergency tracheostomy was performed. Bronchoscopy revealed ulcerative lesions involving the subglottis and vocal cords with overlying white plaques (Figure 2). Histopathological examination of bronchoscopic biopsy specimens showed necrotic tissue infiltrated by septate hyphae with acute‐angle branching on haematoxylin–eosin staining, consistent with tissue invasion by *Aspergillus* species. Fungal culture of the biopsy specimens grew *Aspergillus fumigatus*, confirming the diagnosis. Antifungal susceptibility testing was not performed.

Based on these findings, she was diagnosed with invasive *Aspergillus* tracheobronchitis limited to the subglottic airway. According to the EORTC/MSGERC criteria, this case fulfilled the definition of proven invasive fungal disease, given the histopathological evidence of tissue invasion. The airway phenotype was classified as ulcerative *Aspergillus* tracheobronchitis, supported by the presence of mucosal ulceration with fungal invasion.

Antifungal therapy with voriconazole was initiated intravenously, with a loading dose of 6 mg/kg every 12 h for the first 24 h, followed by a maintenance dose of 4 mg/kg every 12 h. The route was subsequently switched to oral administration as the patient's condition stabilised. Therapeutic drug monitoring was performed to ensure adequate serum trough levels. The intended duration of antifungal therapy was 6 months.

The clinical course was monitored by serial bronchoscopic examinations, which demonstrated gradual regression of the ulcerative lesions and improvement of airway stenosis.

**FIGURE 3:**
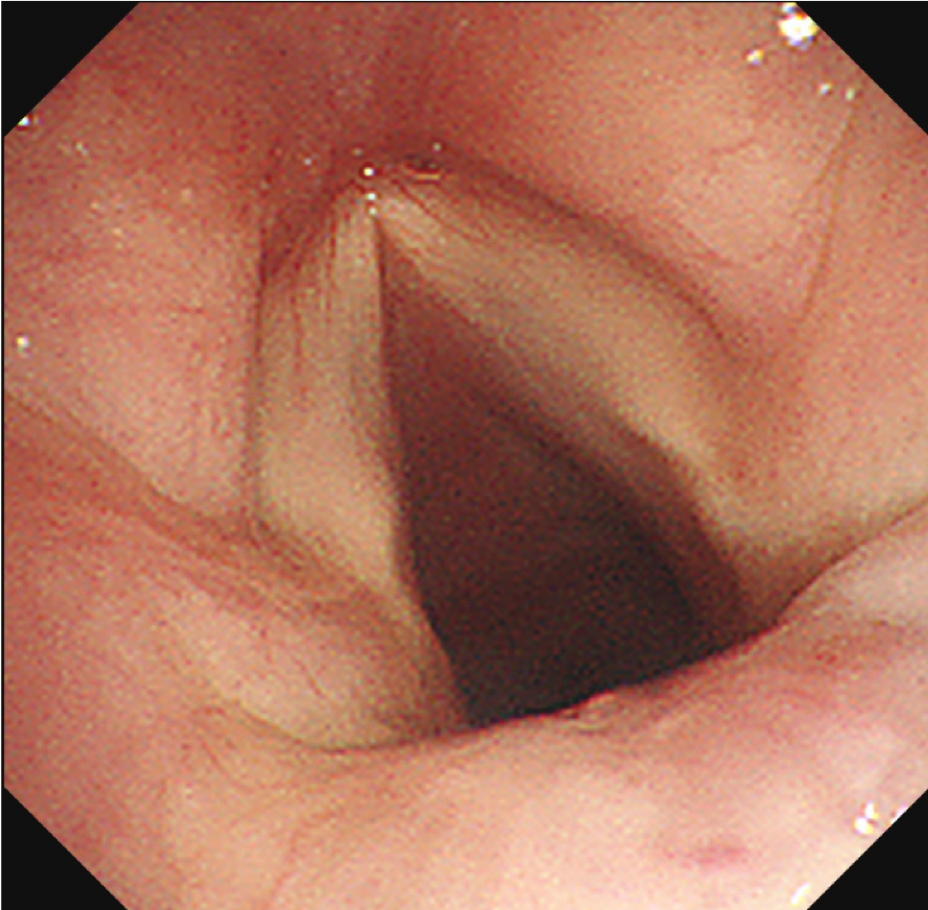
Follow‐up bronchoscopy at 20 weeks showed complete resolution of the subglottic and vocal cord lesions.

The clinical course was monitored by serial bronchoscopic examinations, which demonstrated gradual regression of the ulcerative lesions and improvement of airway stenosis. The tracheostomy tube was successfully removed 10 weeks after initiation of antifungal therapy. She was discharged 11 weeks after admission while continuing antifungal therapy. At 20 weeks, follow‐up bronchoscopy demonstrated complete mucosal healing of the trachea with no residual stenosis (Figure 3).

## 3 Discussion

Invasive aspergillosis remains a significant cause of morbidity and mortality among immunocompromised patients, particularly those with hematologic malignancies or receiving immunosuppressive therapy. Although pulmonary parenchymal involvement is the most common manifestation, IATB is recognised as a rare but important variant of this disease. According to Krenke and Grabczak [3], IATB without associated parenchymal lung disease has been described in only a small number of patients. Reports of IATB confined solely to the subglottic region are extremely limited. In these cases, *Aspergillus fumigatus* is the predominant pathogen, accounting for approximately 63% of infections, whereas *Aspergillus flavus* is less frequently isolated [4].

The pathogenesis of IATB involves direct colonisation of the tracheobronchial tree by inhaled spores that penetrate the mucosal barrier, especially in patients with impaired local or systemic immunity. The airway mucosa, especially at anatomic sites prone to mechanical irritation or reduced clearance, may provide a favourable environment for fungal growth [5]. This is especially relevant in the subglottic region and proximal trachea, where turbulent airflow, foreign‐body aspiration or mechanical injury may contribute to localised infection. Our patient's history of diffuse large B‐cell lymphoma (DLBCL), although in remission, likely posed a residual immunologic risk, predisposing her to opportunistic fungal infection. In our case, histopathological examination demonstrated septate hyphae with acute‐angle branching infiltrating necrotic submucosal tissue on haematoxylin–eosin staining, providing clear evidence of true tissue invasion rather than superficial colonisation. This was further supported by the isolation of *Aspergillus fumigatus* from biopsy specimens obtained from the involved airway.

Denning [5] categorised invasive IATB into three histopathologic subtypes based on the depth of invasion: simple *Aspergillus* tracheobronchitis (superficial colonisation with inflammation and secretions but no mucosal invasion), ulcerative *Aspergillus* tracheobronchitis (characterised by ulceration and histologic evidence of tissue invasion) and pseudomembranous *Aspergillus* tracheobronchitis (marked by extensive endoluminal membrane formation overlying necrotic mucosa). The three forms may coexist in the same patient, and progression from superficial colonisation to deeper tissue invasion is possible, particularly in the setting of severe immunosuppression. Wu and colleagues also proposed a four‐type classification (Types I–IV) based on the extent of involvement: Type I, superficial infiltration of the bronchial mucosa; Type II, full‐layer involvement of the bronchial wall; Type III, occlusive mass formation within the bronchus; and Type IV, a mixed pattern [6]. In the present case, the diagnosis and phenotypic classification were primarily based on Denning's framework, with histopathological confirmation supporting the ulcerative subtype of invasive *Aspergillus* tracheobronchitis.

In our case, the initial bronchoscopic findings with ulceration were consistent with the ulcerative subtype described by Denning. According to Wu's classification, the findings were most consistent with Type II disease. These categorisations underscore the utility of both classification schemes in characterising the spectrum of IATB manifestations. This form carries a worse prognosis than simple colonisation because it represents true tissue invasion, with the potential for vascular infiltration and necrotizing infection. Denning and other authors have emphasised that once the fungal hyphae penetrate the submucosa and vascular structures, patients are at risk for devastating complications, such as bronchovascular fistula formation, massive hemoptysis, and respiratory failure. The mortality rate for IATB has been reported to be 40%–89% [7].

Clinically, IATB presents with nonspecific symptoms such as cough, wheezing, dyspnea and fever, which can mimic bacterial bronchitis, asthma exacerbation or even airway tumours. This often leads to delayed diagnosis, which contributes to the high mortality. In our patient, bronchoscopy identified the subglottic and proximal tracheal lesions and enabled biopsy and culture. Bronchoscopic examination is the diagnostic gold standard for IATB because radiographic imaging can be nonspecific or even normal when parenchymal disease is absent [8]. Histopathological confirmation of hyphal invasion together with positive fungal cultures is essential for a definitive diagnosis.

Another unique aspect of this case is the development of significant subglottic airway stenosis necessitating tracheostomy. Airway narrowing in IATB typically results from a combination of mucosal necrosis, granulation tissue formation and subsequent fibrosis during the healing phase. While ulcerative and pseudomembranous forms of IATB are more likely to cause luminal obstruction, involvement of the subglottic region is particularly problematic because of its narrow diameter and critical role in maintaining airway patency. In our patient, early airway control with tracheostomy prevented severe respiratory compromise and facilitated the delivery of antifungal therapy.

Voriconazole remains the first‐line antifungal agent for invasive aspergillosis and has been associated with improved survival compared with conventional amphotericin B therapy [9]. In this case, prolonged antifungal treatment led to gradual resolution of the necrotic lesion, and serial bronchoscopies confirmed progressive healing. The tracheostomy tube was safely removed after 10 weeks, and the patient remained free of recurrence at follow‐up. This favourable outcome highlights the importance of early recognition, prompt local airway management, and appropriate systemic antifungal therapy.

In conclusion, we report a rare case of subglottic airway stenosis secondary to ulcerative *Aspergillus* tracheobronchitis in a patient with a history of DLBCL. This case adds to the limited literature on IATB involving the proximal trachea and underscores the need for heightened clinical awareness, prompt bronchoscopic evaluation, and multidisciplinary management to prevent life‐threatening complications. Given the persistently high mortality rates associated with IATB, particularly in patients with underlying hematologic malignancies, further studies are needed to better characterise risk factors, optimise diagnostic strategies and develop effective management protocols.

## Ethics Statement

We affirm this research adheres to the ethical standards and guidelines set by Jichi Medical University Saitama Medical Center and has received approval from IRB on 10/July/2025. The IRB approval number is S25‐051.

## Consent

The authors declare that written informed consent was obtained for the publication of this manuscript and accompanying images using the form provided by the Journal.

## Conflicts of Interest

The authors declare no conflicts of interest.

## Data Availability

The data that support the findings of this study are available on request from the corresponding author. The data are not publicly available due to privacy or ethical restrictions.
